# Commentary: A multicenter, real-world cohort study: effectiveness and safety of Azvudine in hospitalized COVID-19 patients with pre-existing diabetes

**DOI:** 10.3389/fendo.2026.1735040

**Published:** 2026-01-20

**Authors:** Shixuan Guo, Jinyuan Zhang, Juan Shu

**Affiliations:** 1 The First School of Clinical Medicine, Lanzhou University, Lanzhou, Gansu, China; 2 The Second School of Clinical Medicine, Lanzhou University, Lanzhou, Gansu,, China; 3 Department of Gerontal Respiratory Medicine, The First Hospital of Lanzhou University, Lanzhou, Gansu,, China

**Keywords:** Azvudine, COVID-19, diabetes, propensity score matching, safety, subgroup analysis

We read with great interest the recent article by Zhang et al. on the effectiveness and safety of Azvudine in hospitalized COVID-19 patients with pre-existing diabetes (1). This multicenter real-world study represents a valuable contribution to the evolving evidence base on antiviral use in high-risk populations. However, several methodological limitations merit attention to better contextualize the findings and guide future research. We wish to offer the following observations.

First, while the application of Kaplan–Meier and Cox proportional hazards regression for time-to-event outcomes is methodologically sound, the analysis does not adequately account for competing risks. Specifically, discharge alive represents a strong competing event that may informatively censor the outcome of in-hospital mortality. The use of cumulative incidence functions and the Fine–Gray model would provide more accurate estimates of absolute risk and avoid potential overestimation of mortality probabilities (2). Additionally, the definition of Azvudine exposure as a time-fixed covariate from admission may introduce immortal time bias, as patients must survive long enough to receive treatment. The analysis does not report the distribution of time from hospital admission to first administration, and immortal time bias cannot be ruled out. Modeling treatment as a time-varying covariate or applying a landmark analysis would help mitigate this bias.

Second, the authors have commendably adjusted for numerous clinical covariates and used propensity score matching to balance baseline characteristics (1). Nevertheless, several potential confounders—including vaccination status (3), smoking (4), and calendar time of admission (5)—were not considered. These factors are plausibly associated with both treatment allocation and COVID-19 outcomes, and their omission may bias the estimated treatment effect. The most consequential sources of unmeasured confounding factor is vaccination status. Whether patients included in the study had received a COVID-19 vaccine prior to illness significantly impacts their final outcomes, thereby further influencing the assessment of mortality rates. Furthermore, the analysis does not account for center-level variability, despite the multicenter design. Incorporating hospital fixed or random effects, or using cluster-robust standard errors, would improve the validity of inferences.

Third, the subgroup analyses are informative but should be interpreted with caution. The authors state that no significant interactions were observed, yet a nominally significant interaction (p=0.044) is reported for HbA1c in the composite outcome (1). This inconsistency, coupled with the absence of multiplicity correction and limited power in some subgroups, underscores the exploratory nature of these findings. Pre-specification of key subgroups and clearer reporting of interaction estimates with confidence intervals would enhance interpretability.

Finally, the safety analysis assessed adverse events from Azvudine initiation until five half-lives after the last dose, but the corresponding risk window for controls was not clearly defined. Asymmetric assessment periods and variable missingness in laboratory data may bias safety comparisons. Standardizing the at-risk period, accounting for informative missingness, and qualifying p-values for multiple testing would strengthen the safety conclusions.

In summary, this study offers important insights into Azvudine use in a high-risk diabetic cohort, but some shortcomings need to be improved and refined. We hope that these analyses and recommendations will be useful for future studies, and together we will promote the progress.
